# The *Lotus japonicus AFB6* Gene Is Involved in the Auxin Dependent Root Developmental Program

**DOI:** 10.3390/ijms22168495

**Published:** 2021-08-06

**Authors:** Alessandra Rogato, Vladimir Totev Valkov, Marcin Nadzieja, Jens Stougaard, Maurizio Chiurazzi

**Affiliations:** 1 Institute of Biosciences and Bioresources (IBBR), CNR, 80131 Napoli, Italy; alessandra.rogato@ibbr.cnr.it (A.R.); vladimir.valkov@ibbr.cnr.it (V.T.V.); 2 Department of Molecular Biology and Genetics, Aarhus University, 8000 Aarhus, Denmark; marcinjn@mbg.au.dk (M.N.); stougaard@mbg.au.dk (J.S.)

**Keywords:** legumes, root elongation, auxin, signaling, nodulation

## Abstract

Auxin is essential for root development, and its regulatory action is exerted at different steps from perception of the hormone up to transcriptional regulation of target genes. In legume plants there is an overlap between the developmental programs governing lateral root and N_2_-fixing nodule organogenesis, the latter induced as the result of the symbiotic interaction with rhizobia. Here we report the characterization of a member of the *L. japonicus TIR1/AFB* auxin receptor family, *LjAFB6*. A preferential expression of the *LjAFB6* gene in the aerial portion of *L. japonicus* plants was observed. Significant regulation of the expression was not observed during the symbiotic interaction with *Mesorhizobium loti* and the nodule organogenesis process. In roots, the *LjAFB6* expression was induced in response to nitrate supply and was mainly localized in the meristematic regions of both primary and lateral roots. The phenotypic analyses conducted on two independent null mutants indicated a specialized role in the control of primary and lateral root elongation processes in response to auxin, whereas no involvement in the nodulation process was found. We also report the involvement of *LjAFB6* in the hypocotyl elongation process and in the control of the expression profile of an auxin-responsive gene.

## 1. Introduction

The major form of auxin in vascular plants, indole-3-acetic acid (IAA), is mainly biosynthesized in aerial tissue and directionally transported to its responsive sink sites, resulting in modulation of local auxin distribution that regulates most aspects of plant growth, development and acclimation [1]. In the root, auxin accumulates at the root tip and afterwards is transported shootward via the lateral root cap and epidermis with a “reverse fountain” transport mechanism [2]. A sophisticated network of signaling pathways is involved in the control of the amount of auxin perceived at the subcellular, cellular, tissue and organ scales.

Changes of auxin levels control cellular responses in part via modulation of transcriptional regulation. Increasing auxin levels promotes physical interactions between Transport Inhibitor1/Auxin-Signaling (TIR1/AFB) receptors and the auxin coreceptors Auxin/Indole-3-Acetic Acid (Aux/IAA) repressor proteins [3,4]. The latter are consequently polyubiquitinated and degraded through the 26S-mediated proteasome action. Degradation of the repressor results in the release of auxin response transcriptional factors (ARFs), which bind to specific Auxin Response Elements (ARE) with the consensus TGTCTC [5], to activate the transcription of the auxin-responsive genes [6,7].

The auxin signaling pathways are extremely diversified in plants, and this may be at least partially due to different combination of TIR1/AFBs and Aux/IAA (29 members in *Arabidopsis thaliana*) proteins characterized by different affinities [8]. The nuclear signaling pathway controlling gene transcription regulates auxin-dependent growth of the shoot toward the light [9] and it is also involved in the root growth inhibition pathway triggered by high auxin concentrations [10,11,12]. However, a more recent model proposes that very rapid auxin perception occurs through an instant cell wall depolarization mediated by calcium influx. This results in root growth inhibition that is then reinforced by the TIR1-dependent transcriptional regulation pathway [10,13,14].

The TIR/AFB family is composed of seven members in *A. thaliana* [15], including the divergent Coronatine Insensitive 1 (AtCOI1), a protein required for efficient response to the plant hormone jasmonic acid [16]. Phylogenetic analysis led to the identification of three pairs of paralogues in *A. thaliana*, TIR1/AFB1, AFB2/AFB3 and AFB4/AFB5 sub-grouped in three distinct clades [17], whereas a fourth clade, AFB6 was lost early in Brassicaceae and Poaceae [17]. Single TIR/AFB mutants have been isolated and phenotypically characterized in *A. thaliana*. *Tir1*, *afb1*, *afb2*, *afb3*, *afb4*, *afb5* mutants appear similar to wild type plants in standard laboratory conditions of growth, whereas these display different level of resistant phenotypes to natural and synthetic auxins. These phenotypes are amplified in double and triple mutant backgrounds, indicating functional redundancy and additive actions [18]. In species other than *A. thaliana*, the investigation of the TIR1/AFB auxin receptor family has been quite limited. Very recently the functional characterization of the *Oryza sativa* TIR1/AFB family was reported [19]. The *O. sativa* members maintain the capacity to physically interact with IAA proteins, and single mutants display a mild resistance to exogenous auxin and partially redundant phenotypes in grain yield, tillering, plant height, root system and germination [19].

In legume plants, most studies have aimed to identify the link between auxin and the nodulation process, whereas the auxin signaling pathways governing root development have been poorly investigated. So far, auxin has been implicated in the control of early steps of nodulation including cell division and rhizobial infection, as well as nodular vascular tissue differentiation [20]. Addition of external auxin at low concentration triggers a slight increase of nodule number in both *L. japonicus* and *Medicago truncatula*-inoculated plants, whereas higher concentrations inhibit nodule formation progressively [21,22]. An inhibitory effect on nodule formation has been also reported by application of transport inhibitors [21,22,23], and all these observations can be associated with the reported physiological local accumulation of auxin at the site of incipient emerging nodule primordium, where cortical cell division take place [23,24,25,26,27]. Auxin biosynthesis and accumulation, induced by perception of rhizobial nodulation (Nod) factors, has been reported in *L. japonicus* root hairs infected by *Mesorhizobium loti* [28]. Furthermore, auxin responses monitored through the analysis of auxin-reporter *GH3* and *DR5*-fused markers were detected in nodule vascular tissues [23,24,26,29,30], and application of auxin transport inhibitors and antagonists led to inhibition of nodule vascular development [23].

Despite the increased amount of information about auxin signal distribution in roots and nodules, relatively little is known about the roles played by the different molecular actors controlling auxin signaling in legume plants. Interestingly, modulation of nodule formation capacity can be triggered in soybean through over-expression of miRNAs controlling the expression of *ARFs* [26,31]. A transcriptomic analysis revealed that different *M. truncatula ARF* genes are induced during the early steps of rhizobial infection, and an insertion mutant of *M. truncatula*, *Mtarf16a*, displayed a reduced number of infection events when compared to controls [27]. The involvement of the TIR/AFB auxin receptors in the nodulation process has been investigated only through RNAi and over-expression approaches to control auxin signaling components in transgenic hairy roots of composite plants [32]. In soybean and *M. truncatula*, over-expression of miRNA393 triggers a reduced expression of *TIR1/AFB* members, leading to hyposensitivity to external auxin [26], which in *M. truncatula* results in reduced nodulation [33].

Here we report the functional characterization of a member of the *L. japonicus TIR1/AFB* auxin receptor family, *LjAFB6*. To our knowledge this is the first protein belonging to the AFB6 clade to be investigated in plants. The analyses of gene expression profiles and the phenotypic characterization of independent null mutants indicate its specialized function in the pathway governing the auxin signaling pathways involved in root development of legume plants.

## 2. Results

### 2.1. Identification of the L. japonicus TIR1/AFB Family

**Figure 1:**
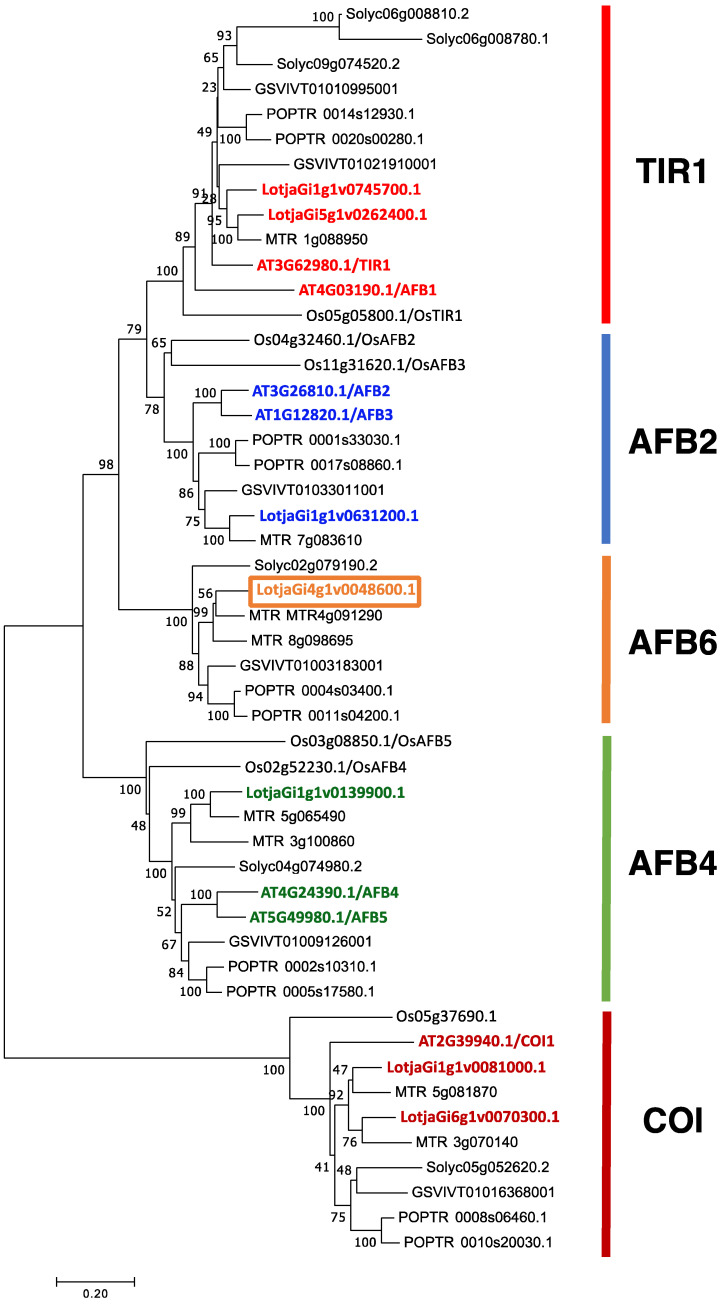
The evolutionary relationship between plant TIR/AFB members. The evolutional history was inferred using the Neighbor-Joining method [39]. The optimal tree with the sum of branch length = 8.90683588 is shown. The tree is drawn to scale, with branch lengths in the same units as those of the evolutionary distances used to infer the phylogenetic tree. The evolutionary distances were computed using the JTT matrix-based method and are in the units of the number of amino acid substitutions per site. The five distinct clades of the TIR/AFB family are indicated by different bar colours on the right. *L. japonicus* and *A. thaliana* members are highlighted by colours. The LjAFB6 member is framed. Abbreviations: Lotja, *Lotus japonicus*; AT, *Arabidopsis thaliana*; MTR, *Medicago truncatula*; Os, *Oryza sativa*, Solyc, *Solanum lycopersicon*; GSVIV, *Vitis vinifera*; POPTR, *Populus trichocarpa*.

In order to explore and compare the role played by auxin signaling pathways on root and nodule development in legume plants, we retrieved the *L. japonicus* TIR1/AFB family through a blast search of the *Lotus japonicus* genome (http://www.kazusa.or.jp/lotus/summary3.0.html (accessed on 1 July 2021); https://lotus.au.dk/ (accessed on 1 July 2021) [34,35], using as queries the *A. thaliana* TIR1/AFB sequences. In *A. thaliana* a small subfamily of seven genes was identified among the 700 F-box-containing proteins, including the divergent Coronatine Insensitive 1 (AtCOI1) [15,16]. We found an equivalent group of seven F-box containing members, sharing a significant level of amino acid identities in both the *L. japonicus* GIFU and MG20 accessions. Significant pair-wise similarities with the *A. thaliana* proteins were scored for the seven *L. japonicus* proteins (Table S1). The phylogenetic relationship of the TIR1/AFB members identified in different plant genomes was obtained and the resulting tree subgrouped in the five clades previously identified in vascular plants (Figure 1) [17].

The seven *L. japonicus* proteins are distributed in the five clades with two members located in the TIR1 and COI clades (Figure 1). LotjaGi4g1v0048600.1 falls in the clade AFB6, where no *A. thaliana* sequences are found, as this clade was lost early in the brassicaceae family [17]. To the best of our knowledge, no members of the AFB6 clade have been characterized in plants, and we started our characterization of the *L. japonicus* family with LotjaGi4g1v0048600.1, hereafter termed *LjAFB6*. *LjAFB6* has a predicted sequence containing three exons and two introns, encoding for a 623 amino acid protein with a predicted molecular mass of 70.05 kDa. Residues involved in the protein AtTIR1 protein oligomerization and auxin binding [36], are conserved in LjAFB6 and highlighted in Figure S1A as well as the N-terminal F-box motif (amino acids 22–60) that is required for the assembly of the F-box protein onto the core of the SCF complex. The three-dimensional structure model obtained using as template the X-ray structure of *A. thaliana* TIR1 (PDB code:2P1M) [37] is shown in Figure S1B.

### 2.2. Regulatory Profiles of Transcription

**Figure 2:**
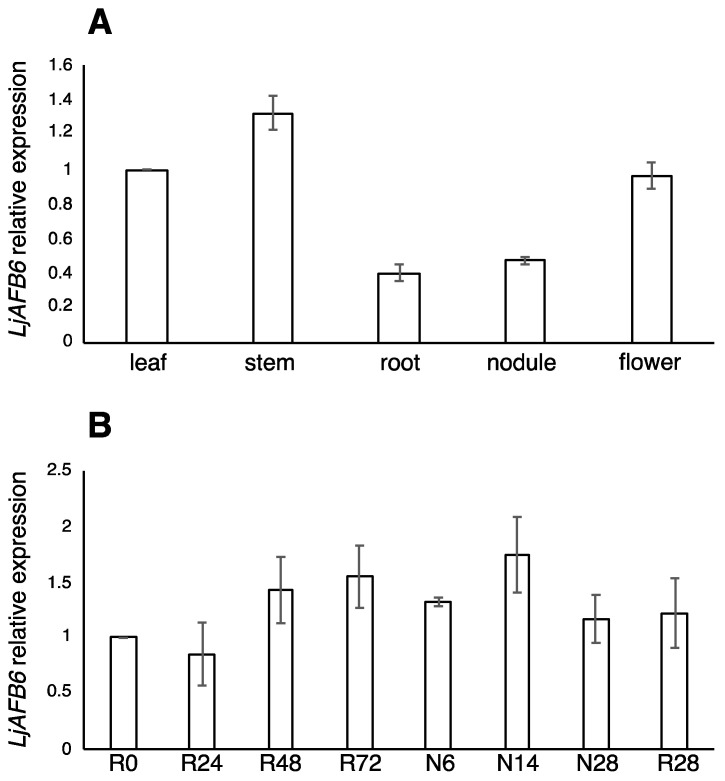
(**A**) *LjAFB6* expression in different organs. RNAs were extracted from wild-type plants grown on Gamborg B5 derivative medium without nitrogen source at 3 weeks post inoculation (wpi). Mature flowers were obtained from *L. japonicus* plants propagated in the growth chamber. (**B**) Time course of expression of *LjAFB6* in root and nodule tissues after *M. loti* inoculation. RNAs were extracted from roots of wild-type seedlings grown in nitrogen starvation conditions at different times after inoculation (R0, 24 h, 48 h, 72 h, 28 days), from nodule primordia (6 days) and mature nodules (14 days and 28 days post inoculation). Expression levels are normalized with respect to the internal control *UBIQUITIN* (*UBI*) gene and plotted relative to the expression of leaf (**A**) and R0 (**B**). Data bars represent means and SD of data obtained with RNA extracted from three different sets of plants and three qRT-PCR experiments.

Expression data retrieved from the Lotus gene atlas (https://lotus.au.dk/expat/ (accessed on 1 July 2021)) [38] indicate a low level of transcriptional regulation in different organs for the seven *L. japonicus TIR1/AFB* genes with a preferential expression in the aerial portions of the plant (Figure S2). In particular, none of the five *Lotus TIR/AFB* auxin receptors genes display a significant up or down-regulated expression in the nodule organ as compared to roots (Figure S2). To investigate the physiological function of the *LjAFB6* gene and to confirm the expression data retrieved from the *L. japonicus* gene atlas, we analyzed the *LjAFB6* transcript abundances in different organs of *L. japonicus* by qRT-PCR. Seedlings germinated on the Gamborg-B5 derived medium without nitrogen sources were inoculated with *M. loti* and amount of *LjAFB6* transcript tested on RNAs extracted from different organs at three weeks after inoculation. The *LjAFB6* gene was mainly expressed in shoot tissues (leaf and stem) and was not significantly modulated in root and nodule organs (Figure 2A). The lack of transcriptional regulation during the nodule organogenesis process was also confirmed in a time course experiment where we compared the *LjAFB6* expression in uninoculated and inoculated roots (Figure 2B).

**Figure 3:**
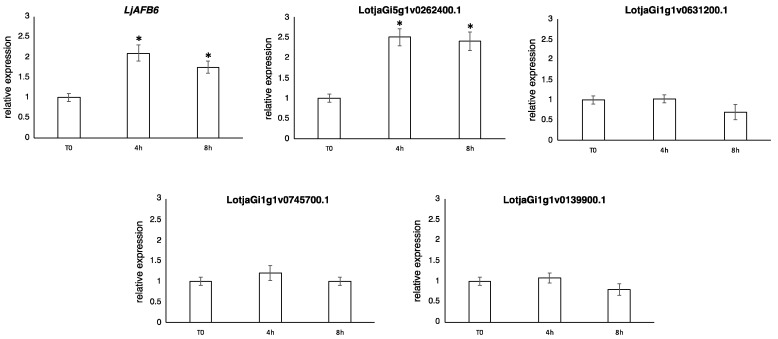
Nitrate dependent expression of the *LjTIR1/AFB* genes. RNAs were extracted from roots of wild-type plants grown for 10 days on Gamborg B5 derivative medium with glutamine 1 mM as the sole nitrogen source and transferred on 1 mM KNO_3_ for the indicated range of times. Expression levels are normalized with respect to the internal control *UBIQUITIN* (UBI) gene and plotted relative to the expression of T0. Data bars represent means and SD of data obtained with RNA extracted from three different sets of plants and three qRT-PCR experiments. Asterisks indicate significant differences with T0 levels. * *p* < 0.01.

In order to investigate a distinctive transcriptional regulatory trait reported for the *A. thaliana TIR1/AFB* family [40,41], we also analyzed the expression profiles of the *L. japonicus* members in response to nitrate supply. *L. japonicus* wild type plants grown for 10 days on B5-derived medium with glutamine 1 mM as the sole nitrogen source were transferred for 4 and 8 h on B5-derived medium with 1 mM KNO_3_ as sole nitrogen source and the *LjTIR1/AFB* transcript levels were tested in the roots by qRT-PCR (Figure 3). An increased amount of transcript after nitrate supply (about 2.5 fold) was identified for two out of the five members of the *L. japonicus* family, *LjAFB6* and *LotjaGi5g1v0262400.1*. However, as control plants were not transferred on new plates with glutamine at four and ten hours, we cannot completely rule out the possibility that the observed changes of expression were due to the experimental manipulation and plant perturbation.

### 2.3. Spatial Profile of LjAFB6 Expression and Protein Subcellular Localization

**Figure 4:**
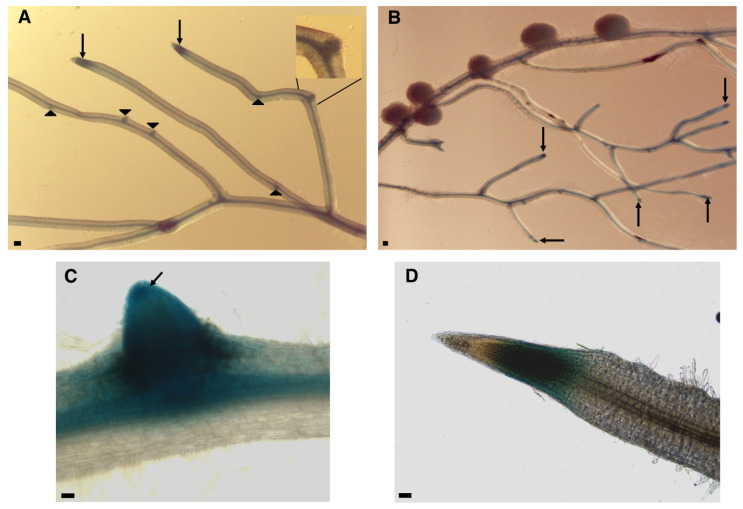
Representative β-glucuronidase (GUS) activity in *L. japonicus* hairy roots transformed with the pr*LjAFB6*-*gus*A construct. (**A**,**B**) Arrows and arrowheads indicate staining in the meristems of primary/lateral elongated roots and in the lateral root primordia, respectively. (**C**) GUS activity in the root stele, in the emergence region of the lateral root and in the apical meristem (arrow). (**D**) GUS activity in the apical region of a primary root. Black bars on the left = 50 μm.

To gain further information about the profile of *LjAFB6* expression in roots and nodules, the PCR fragment containing the 5′ region of the gene extending up to 1222 bp upstream of the ATG and including the first 11 *LjAFB6* codons, was subcloned in the pBI101.1 binary vector to obtain a translational fusion with the *gus*A reporter gene [42]. *Lotus* composite plants obtained upon transformation with *Agrobacterium rhizogenes* were inoculated with *M. loti* and GUS activity tested in hairy roots. Strong GUS activity was detected in the apical meristem of primary and elongated lateral roots and in the developing lateral root primordia (Figure 4A–D). Staining was also detected with lower intensity in root vascular bundle (Figure 4A,B), whereas no activity could be observed in mature nodules (Figure 4B).

To determine the subcellular localization, we generated a construct that fused YFP to the C-terminus of LjAFB6 under the control of the cauliflower mosaic virus 35S promoter and introduced this construct into tobacco protoplasts. Confocal microscopy indicated that the 35S-LjAFB6-YFP fusion was exclusively localized to the nucleus of transformed protoplasts (Figure S3A–C).

### 2.4. Isolation of LORE1-Insertion Null Mutants and Root Elongation Phenotypic Analyses

**Figure 5:**
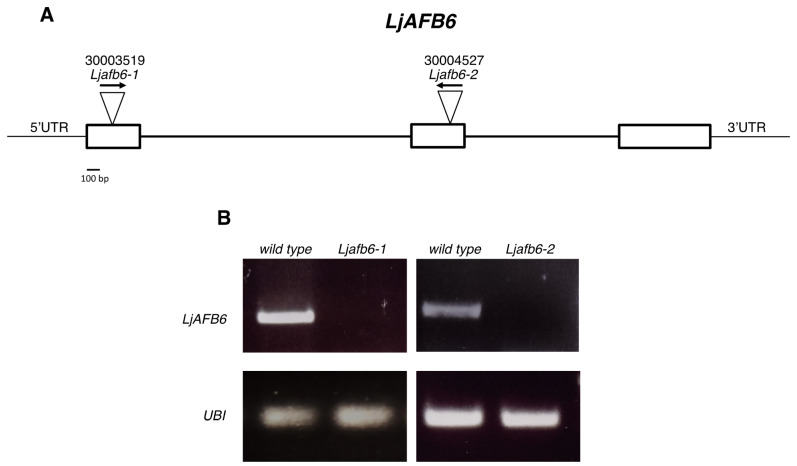
Structure of the *L. japonicus AFB6* gene and analysis of the expression in the LORE1 segregants. (**A**) Gene model of *LjAFB6*. Insertion sites and relative orientations of the LORE1 retrotransposon element in the 30003519 and 30004527 lines are indicated. (**B**) Expression of the *LjAFB6* gene in the *Ljafb6-1* and *Ljafb6-2* plants. Total RNAs isolated from leaves was used for qRT-PCR analyses.

To investigate the involvement of *LjAFB6* in the auxin response, we isolated two independent LORE1 insertion mutants from the *L. japonicus* GIFU LORE1 collection lines [43,44,45]. Lines 30003519 and 30004527, bearing retrotransposon insertions in the first, and second exon (Figure 5A), were genotyped by PCR to identify homozygous plants for the insertion events. Endpoint RT-PCR analyses of homozygous plants from lines 30003519 and 30004527 revealed no detectable *LjAFB6* mRNA in shoots, and hence were considered null mutants and hereafter called *Ljafb6-1* and *Ljafb6-2*, respectively (Figure 5B). Two individual homozygous mutant lines for both LORE1 insertions were selected for analyses, and because their growth phenotypes did not significantly differ, the data obtained with the selected individual mutants were pooled in the phenotypical analyses.

**Figure 6:**
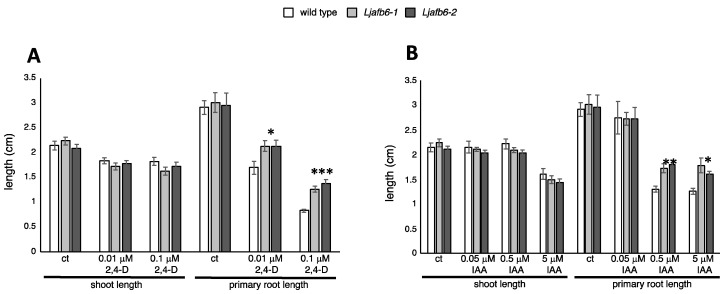
Phenotypic characterization of *Ljafb6-1* and *Ljafb6-2* mutants. Wild type and *Ljafb6* plants were grown in the presence of different concentrations of 2,4-D and IAA. (**A**) Shoots and primary roots length of wild type and *Ljafb6* plants, 10 days after transfer on 2,4-D media. (**B**) Shoots and primary roots length of wild type and *Ljafb6* plants 10 days after transfer on IAA media. Ct indicates control plants grown without addition of hormones. Bars represent means and SE of measures from three experiments (12 plants per experiment per condition). Asterisks indicate significant differences between plant genotypes in the same conditions of growth. * *p* < 0.05; ** *p* < 0.002; *** *p* < 0.0001.

**Figure 7:**
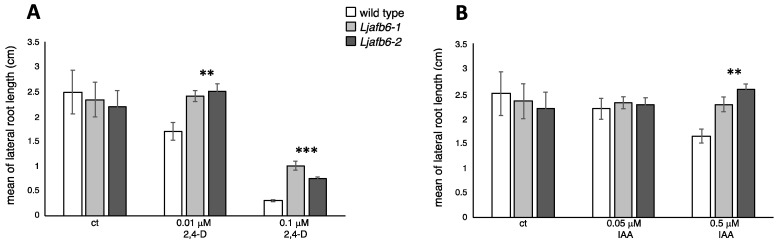
Phenotypic characterization of *Ljafb6-1* and *Ljafb6-2* mutants. (**A**) Mean of lateral roots length of wild type and *Ljafb6* plants 20 days after transfer on 2,4-D media. (**B**) Mean of lateral roots length of wild type and *Ljafb6* plants 20 days after transfer on IAA media. Bars represent means and SE of measures from three experiments (12 plants per experiment per condition). Asterisks indicate significant differences between plant genotypes in the same conditions of growth. ** *p* < 0.002; *** *p* < 0.0001.

The auxin signaling pathways controlling root elongation have not been studied adequately in legume plants as most of the studies have been focused on the correlation with the nodulation process [22,27,46]. With only limited information describing the experimental conditions to highlight the root response to external hormone applications in the *Ljafb6* genetic background, we first determined the optimal range of concentrations. In *A. thaliana*, the root elongation phenotype in response to external auxin has been mainly tested with the synthetic auxin 2,4-D provided at concentrations ranging from 0.01 to 0.15 μM [17,18,36,47,48]. Therefore, three days old wild type synchronized *L. japonicus* seedlings with comparable primary root lengths were transferred on B5 medium supplemented with 0.01 μM and 0.1 μM 2,4-D to test the effects on shoot and root elongation up to 20 days from the transfer (Figure 6 and Figure 7). The primary root length of wild type plants, scored at 10 days, displayed a progressive decrease in the presence of 0.01 μM and 0.1 μM 2,4-D as compared to control plants, whereas the shoot elongation was slightly inhibited by the addition of the synthetic auxin (Figure 6A).

The emerged lateral roots were still too short to be feasibly measured at 10 days of exposure to auxin, and the data reported in Figure 7 refer to the scoring of the lateral root length at 20 days. The wild type lateral roots displayed a clear-cut progressive reduction of length in the presence of 0.01 μM and 0.1 μM 2,4-D (Figure 7A and Figure S4A–F).

As the effects triggered on plant growth by the external addition of natural and synthetic auxins may differ each other quantitatively and qualitatively, we also scored the *L. japonicus* root response phenotype after exposure to a wide range of IAA concentrations. The lowest 0.05 μM concentration did not affect significantly the root architecture response in wild type *L. japonicus* plants (Figure 6B and Figure 7B), whereas the presence of 0.5 μM and 5 μM IAA caused a significant reduction of the primary root length, scorable at 10 days after the exposure (Figure 6B). At 5 μM IAA concentration we observed a significant reduction of the shoot elongation response (Figure 6B). The lateral roots elongation was also significantly reduced at 0.5 μM IAA, although not as severely as in the presence of 2,4-D (Figure 7B).

Interestingly, primary and lateral root elongation responses of both *Ljafb6* mutants were also progressively affected by increasing concentrations of 2,4-D and IAA, but these effects were significantly reduced compared to wild type plants (Figure 6 and Figure 7). In particular, the *Ljafb6* primary roots were about 20% (0.01 μM 2,4-D) and 38% (0.1 μM 2,4-D) longer than wild type after 10 days of exposure (Figure 6A). The same phenotypic response was also displayed by the *Ljafb6* plants after 10 days of exposure to 0.5 μM IAA and 5 μM IAA (Figure 6B). Concerning the lateral root elongation, the average increase scored after 20 days of exposure to 0.01 μM 2,4-D and 0.1 μM 2,4-D, was about 31% and 65% in the *Ljafb6* mutants as compared to wild type plants, respectively (Figure 7A and Figure S4A–F). A reduced response of the lateral root elongation phenotype in the *Ljafb6* genotype, was also observed in the presence of 0.5 μM IAA (Figure 7B; Figure S5A,B).

These results demonstrate an alteration of the auxin-dependent responses in the *Ljafb6* genetic background. Furthermore, it is remarkable that, although expected, growth in the presence of auxin stimulated lateral root formation, we did not observe in the *L. japonicus afb6* mutants, the reduced density of lateral roots reported for the *A. thaliana tir1* and *afb1* mutants [3,17] (Figure S6).

### 2.5. LjAFB6 Is Involved in the Hypocotyl Elongation Process

**Figure 8:**
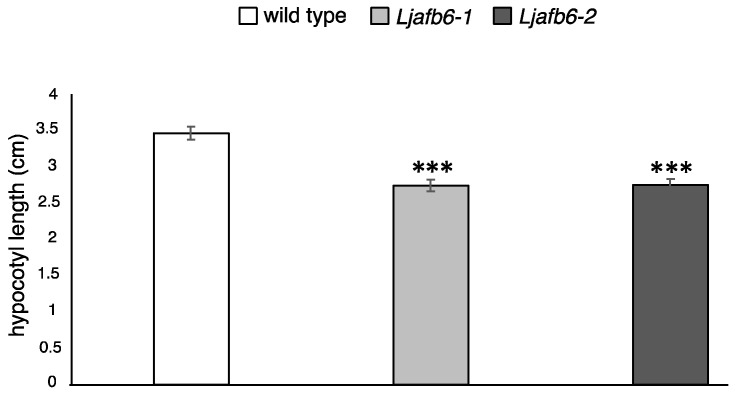
Phenotypic characterization of *Ljafb6-1* and *Ljafb6-2* mutants. Hypocotyl length of wild type and *Ljafb6* etiolated seedlings maintained for 2 weeks in the dark. Asterisks indicate significant differences between plant genotypes. *** *p* < 0.0001.

We also compared another auxin-dependent phenotype represented by the hypocotyl elongation. Etiolated wild type and mutant seedlings were obtained after germination in the dark for two weeks at 23 °C. As expected, a dramatic increase of the hypocotyl length was observed for both genotypes, but reduced length of the hypocotyl was scored in the mutant seedlings (Figure 8; Figure S5C). The causal relationship between KO mutations in the *LjAFB6* gene and the reported phenotypes were confirmed by cosegregation analyses conducted on the progeny of plants heterozygous for the LORE1 insertion event into the *LjAFB6* gene. All the segregants genotyped as homozygous for the insertion event showed the short hypocotyl phenotype, whereas such a phenotype was not exhibited by heterozygous and wild type plants.

### 2.6. Auxin Responsive Profiles of Expression

**Figure 9:**
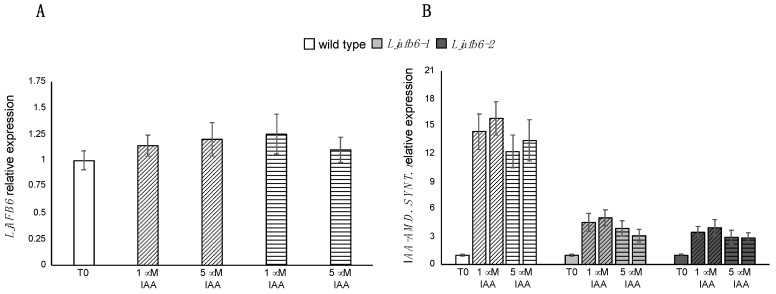
Auxin responsive profiles of expression. (**A**) Wild type plants were treated with 1 and 5 μM IAA for 12 and 24 h. (**B**) Wild type and *Ljafb6* plants were treated with 1 and 5 μM IAA for 12 and 24 h. Genotypes and growth conditions are indicated. Bars with oblique and horizontal motifs indicate 12 h and 24 h samples, respectively. RNAs were extracted from roots and qRT-PCR performed using primers listed in Table S2. Expression levels were normalized with respect to the internal control *UBIQUITIN* (*UBI*) gene and plotted relative to the expression of T0. Data bars represent means and SD of data obtained with RNA extracted from three different sets of plants and three qRT-PCR experiments.

The phenotypic characterization of the two null mutants clearly indicated the contribution of *LjAFB6* to auxin responses in both primary and lateral root elongation processes (Figure 6 and Figure 7). Therefore, to determine whether auxin regulates expression of the *LjAFB6* gene, we analyzed its expression in *L. japonicus* wild type roots exposed for 12 and 24 h to 1 and 5 μM IAA. The results shown in Figure 9A indicate no changes of the *LjAFB6* transcript level under both IAA treatments. We have further tested the involvement of *LjAFB6* in auxin response signaling by comparing the expression of a *GRETCHEN HAGEN 3* (*GH3)* auxin-responsive gene in the wild type and *Ljafb6* genetic backgrounds. The profiles of expression of the IAA *Amido Synthetase* (LotjaGi2g1v0405900.1) were analyzed in roots of wild type and *Ljafb6* plants, transferred at ten days after sowing, on a medium supplemented with 1 μM and 5 μM IAA. The early transcriptional induction of this ARF marker in the roots of *L. japonicus* plants exposed to IAA was previously reported [49]. The transcription of the *GH3* auxin responsive gene was strongly induced at both IAA concentrations, and the level of induction was reduced of about four-fold in the *Ljafb6-1* and *Ljafb6-2* mutants compared to wild type (Figure 9B).

### 2.7. Analyses of the Symbiotic Phenotypes

**Figure 10:**
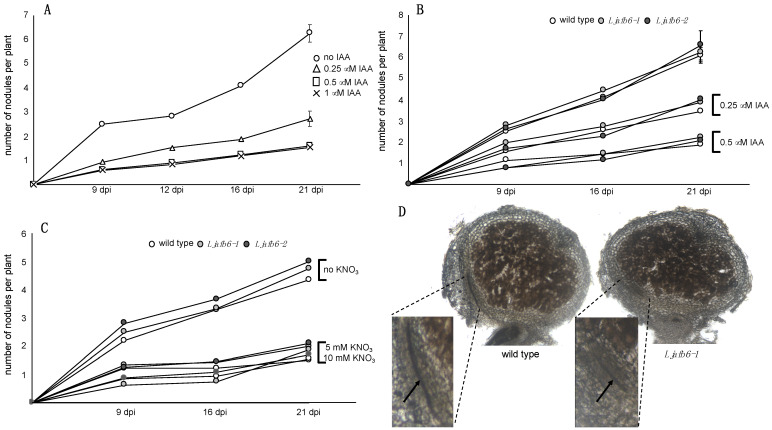
Symbiotic phenotypes of wild type and *Ljafb6* plants. (**A**) Kinetic of nodulation in wild type plants grown in the presence of different concentrations of IAA. IAA concentrations are indicated. (**B**) Kinetic of nodulation in wild type and *Ljafb6* plants grown in the presence of 0.25 and 0.5 μM IAA. (**C**) Kinetic of nodulation in wild type and *Ljafb6* plants grown in permissive conditions (without nitrate) and inhibitory conditions (5 and 10 mM KNO_3_). Nodules were scored weekly after inoculation, up to three weeks. KNO_3_ concentrations are indicated. (**D**) 80 μm vibratome sections of wild type and *Ljafb6-1* nodules. Higher magnification images refer to zones of the nodular sections where vascular bundle structures are visible (arrows).

In order to test whether *LjAFB6* is also involved in the auxin signaling pathways controlling different steps of the nodulation process, we set up the conditions for the symbiotic phenotypic characterization by identifying the range of auxin concentrations needed to highlight symbiotic phenotypes in our experimental conditions. Addition of IAA at low concentration (0.01 μM) has been reported to trigger a slight increase of nodule number in both *L. japonicus* and *M. truncatula* inoculated plants, whereas higher concentrations (0.2–1 μM) inhibited nodule formation progressively [21,22]. Synchronised *L. japonicus* wild type seedlings were transferred 6 days after sowing on B5 Gamborg-derived medium without nitrogen sources supplied with different IAA concentrations ranging from 0.25 μM to 1 μM and inoculated with *M. loti*. The inhibitory effect of 0.25 μM IAA resulted in halving the nodule formation capacity and this action was further increased at 0.5 and 1 μM (Figure 10A). Therefore, we compared the nodulation capacity of the different *L. japonicus* genotypes in the presence of 0.25 and 0.5 μM IAA. No significant difference was observed (Figure 10B). Furthermore, in agreement with the profile of expression shown in Figure 1A,B and Figure 2B, we did not detect any difference in the development of mature nodule and vascular bundle structures (Figure 10D). Noteworthy is the confirmation of increased lateral root elongation displayed by the *Ljafb6* plants in the presence of 0.25 μM and 0.5 μM IAA compared to wild type plants (Figure S7). Finally, in order to investigate the possible involvement of *LjAFB6*, whose expression is induced by KNO_3_ (Figure 3), in the cross-talk between nitrate and auxin signals controlling the nodulation process, we tested whether the nitrate-dependent inhibitory pathway triggered by high external KNO_3_ concentration was altered in the *Ljafb6* backgrounds. Synchronized wild type and *Ljafb6* seedlings were transferred after germination in Petri dishes with B5 derived media without nitrogen sources or in the presence of 5 and 10 mM KNO_3_ and inoculated with *M. loti*. As shown in Figure 10C the curve of nitrate-dependent nodulation inhibition is conserved among the different genotypes.

## 3. Discussion

In legume plants unraveling the roles played by factors involved in the auxin signaling pathways governing root and nodule development is crucial to establish functional boundaries between two organogenic processes that display similarities [24,50,51,52]. Both lateral roots and nodule development are regulated by a complex hormonal network, with the auxin involved in the control of organ primordia initiation, emergence and vasculature differentiations. We report here the characterization of a member of the *L. japonicus* TIR1/AFB family based on the analyses of temporal and spatial profiles of expression and phenotypes of independent null mutants.

The spatial pattern of expression exhibited by the pr*LjAFB6-gus*A fusion in the root tissues, with GUS activity detected in the meristematic regions of primary and lateral roots as well as lateral root primordia and vascular structures (Figure 4A–D) and the reported LjAFB6 nuclear localization (Figure S3), are comparable to results reported for most of the *A. thaliana TIR1/AFB* genes [17,18,53]. In legume plants, a similar pattern of promoter activity has been reported for the four *GmTIR1A/B/C/D* and the two *GmAFB3A/B* predicted genes retrieved in the allotetraploid soybean genome [32]. In particular, in soybean hairy roots GUS activity was displayed in the main and lateral root tips and lateral root primordia, with weaker GUS staining exhibited by the pr*GmAFB3-gus*A fusion [32]. Interestingly, the root meristem and columella cell root cap expression were also reported for an auxin-responsive DR5 promoter fusion in *M. truncatula* [26]. The preferential GUS expression exhibited in the primary and lateral root tips is consistent with *LjAFB6* involvement in the auxin-dependent signaling pathways governing root elongation. All the *A. thaliana* TIR1/AFB members were demonstrated to act as auxin receptors by promoting the degradation of the Aux/IAA transcriptional repressors resulting in the release of the ARFs transcriptional factors. The involvement of *LjAFB6* in this auxin response signaling pathway was confirmed by the significantly reduced expression of the *GH3* auxin responsive gene in the roots of *Ljafb6* as compared to wild type plants grown in the presence of IAA (Figure 9B). The lack of the transcriptional *LjAFB6* auxin responsiveness reported in Figure 9A is also consistent with the expression profiles reported for the *A. thaliana* *TIR1/AFB* genes [17], but different from responses observed in soybean, where *GmTIR1A*, *GmTIR1C* and *GmAFB3A* are induced by 2,4-D treatment in roots [32].

The main phenotypic trait reported in the *A. thaliana tir1*, *afb1*, *afb2*, *afb3*, *afb4* and *afb5* single mutant backgrounds was increased primary root growth in the presence of auxin, whereas they appeared similar to wild type plants in standard laboratory conditions of growth [11,17,18,36,47,48,53]. An increased root elongation tolerance in the presence of 2,4-D was recently reported also in *O. sativa tir1/afb* single mutants [19].

In legumes, the experimental setup for testing root responsiveness to phytohormone treatments is not an easily predictable matter, as those physiological traits that have been associated to the acquisition of symbiotic predisposition appear to be peculiar in this plant family [54]. Therefore, the optimal range of 2,4-D and IAA concentration exploited to highlight clear-cut root phenotypes as well as the timing of the plant exposure to the hormone had to be tailored for *L. japonicus* root responsiveness. In particular, *L. japonicus* primary roots displayed a sensitivity response to 0.01 μM and 0.1 μM 2,4-D similar to those reported in *A. thaliana* (Figure 6A), although in the latter a short time of exposure was exploited for scoring the root elongation phenotype [17,18,36,47,48]. On the other side, *L. japonicus* primary roots appeared to be more tolerant than *A. thaliana* to IAA, where 25% and 50% growth inhibition were reported in the presence of 0.03 μM and 0.1 μM concentrations (Figure 6B). However, these quantitatively different primary root responses need to be further investigated to narrow the curve of sensitivity.

Importantly, the analyses conducted on the two *Ljafb6* knock out LORE1 mutants revealed a significant increased tolerance of the primary root growth in plants exposed for 10 days to 2,4-D and IAA as compared to wild type plants (Figure 6A–D). Furthermore, etiolated mutant seedlings displayed a significant hypocotyl length reduction compared to wild type seedlings, indicating the involvement of *LjAFB6* in another auxin dependent elongation process (Figure 8). A similar phenotype was reported in *A. thaliana* only for *Attir1* single mutants that displayed a short hypocotyl length compared to wild type after incubation at 28 °C [11,47]. Therefore, our analysis reveals that a member of the AFB6 clade such as *LjAFB6*, apparently shares the involvement in the primary root elongation response to auxin reported for the *A. thaliana* and *O. sativa TIR1/AFB* genes. Furthermore, a very interesting phenotype revealed by the comparison of *Ljafb6* and wild type plants is the striking increased tolerance of the lateral root elongation to both 2,4-D and IAA in the mutated genotypes (Figure 7; Figures S4–S6). These results suggest a functional correlation of the *LjAFB6* and *AtAFB3* genes, as the latter was the only member of the *A. thaliana* family to display alterations of both primary and lateral root growth in the mutated background [40]. *AtAFB3*, whose nitrate-dependent induction of expression was correlated in the root tips with an increased auxin activity, has been reported to be a master regulator of the nitrate dependent signaling cascade controlling the root system architecture [40,41]. This nitrate-dependent pathway depends on the nitrate transport function of AtNPF6.3, a nitrate transporter that also functions as an auxin transport facilitator [55,56] and acts through the transcription factor *AtNAC4* target [40,41,57,58].

In the case of *LjAFB6*, the correlation with a nitrate dependent signaling was suggested by the transcriptional induction shown in Figure 3, where a transient peak of expression is observed at 4 h after the transfer in 1 mM KNO_3_ conditions, a profile very similar to the one reported for *AtAFB3* [40]. However, the involvement of *LjAFB6* in a nitrate dependent signaling pathway controlling root architecture must be further investigated because the main phenotypic trait reported for *Atafb3*, which is the rescue of the inhibitory effect exerted by high concentration of nitrate (5 mM) on root development [40], could not be tested in the *Ljafb6* background since such an inhibitory effect of nitrate on root development in *L. japonicus* plants has never been reported, even in the presence of 20 mM KNO_3_ [59].

On the other hand, a nitrate dependent signaling pathway responsible of the inhibition of nodule organogenesis in the presence of external high concentrations of KNO_3_ is well-known for legume plants [60,61,62], but the result shown in Figure 10C with the overlapping curves of inhibition of nodulation in the presence of progressively increased concentrations of KNO_3_ in wild type and *Ljafb6* plants, seems to exclude this functional link. A functional link of *LjAFB6* with the nodulation process is also excluded by the profile of expression of *LjAFB6* shown in Figure 2A,B, which did not indicate a transcriptional regulation temporally associated with root infection, nodule development or functioning processes. This unregulated pattern of expression is shared with all the other members of the *Lotus* family (Figure S2). Consistently, the spatial profile of expression shown in Figure 4, rules out the possible involvement of *LjAFB6* in the nodulation process, as GUS activity was not detected in nodules developed in the inoculated hairy roots transformed with the pr*LjAFB6*-*gus*A fusion (Figure 4B). The phenotypic characterization reported in Figure 10 further confirmed that conclusion, as no differences were scored between wild type and nodule plants for the nodule formation capacity, as well as nodule development with and without addition of auxin in the media. These results indicating the lack of any obvious functional link with the nodulation process for *LjAFB6* are distinct from the ones reported in soybean for the four *GmTIR1* and *GmAFB3* predicted sequences, which displayed significant promoter activity in the nodules of the inoculated hairy roots, with the exception of *GmAFB3B* that did not exhibit a significant expression [31]. Their potential roles in the perception of auxin during nodulation in soybean were confirmed by reduced or increased nodule numbers displayed by hairy roots transformed with *GmTIR1A*, *GmTIR1B*, *GmAFB3A* RNAi and over-expressing constructs, respectively [31]. However, a complete characterization of the *L. japonicus TIR1/AFB* family is necessary to establish putative orthologous relationships between the different members in the two legume plants.

## 4. Materials and Methods

### 4.1. Plant Material and Growth Conditions

All experiments were carried out with *Lotus japonicus* ecotype B-129 F12 GIFU [63,64]. Plants were cultivated in a controlled growth chamber with a light intensity of 200 μmol m^−2^ s^−^^1^ at 23 °C with a 16 h:8 h, light:dark cycle. Seeds sterilization was performed as described in [65]. Five days after sowing in axenic conditions on H_2_O agar Petri dishes, unsynchronized seedlings were discarded. Seedlings were transferred on sterile filter paper placed on agar slants of B5 [66] or B5 derived growth media with/out 2,4-D (D8407; Sigma Aldrich, St. Louis, MO, USA) and IAA (I0901; Duchefa, Haarlem, The Netherlands). Filter papers (070115; DIAPATH) with growing plants were transferred on fresh media with/out hormones every 8 days. The root system was shielded from light access (even overhead light) by fitting the plates into a blacked-out container and by shielding the roots with autoclaved aluminium foil. *Mesorhizobium loti* inoculation was performed at 7 d after sowing as described in [67]. The strain R7A used for the inoculation experiments was grown in liquid TYR-medium supplemented with rifampicin (20 mg L^−1^). In the case of *M. loti* inoculation plants were transferred on medium with the same composition as Gamborg B5 medium [66], except that (NH_4_)_2_SO_4_ and KNO_3_ were omitted. KCl was added, when necessary, to the medium to replace the same concentrations of potassium source. The media containing vitamins (G0415; Duchefa) were buffered with 2.5 mM 2-(N-morpholino)ethanesulfonic acid (MES, M1503.0250; Duchefa) and pH-adjusted to 5.7 with KOH. Plant length parameters were measured with the ImageJ software [68].

### 4.2. Lotus Japonicus Transformation Procedures

Binary vectors were conjugated into the *Agrobacterium rhizogenes* 15834 strain [69]. *A. rhizogenes*-mediated *L. japonicus* transformations and inoculation of composite plants were performed as described in [70].

### 4.3. Protoplast Transformation

Leaf protoplasts were prepared and transformed according to [71] using 3-wk-old *N. tabacum* plants. DNA (40 μg of each construct) was introduced into 1 × 10^6^ protoplasts by polyethylene glycol (PEG)-mediated transfection. After 16 h incubation in the dark at 25 °C, yellow fluorescent protein (YFP) fluorescence in protoplast cells was detected by confocal microscopy.

### 4.4. Plasmids Preparation

pr*LjAFB6-gus*A, the PCR-amplified fragment containing 1222 bp upstream of the ATG, was obtained on genomic DNA with forward and reverse oligonucleotides containing *Sal*I and *Bam*HI sites, respectively (Table S2). The amplicon was subcloned as *Sal*I-*Bam*HI fragment into the pBI101.1 vector [42] to obtain the T-DNA construct. Finally, the pr*LjTIR1/AFB-gus*A cassette was subcloned as an *Eco*RI–*Hind*III fragment into the pIV10 plasmid for cointegration into the pAR1193 [69]. LjAFB6-YFP, the PCR-amplified fragment obtained on leaf cDNA with forward and reverse oligonucleotides containing *BglII* and *Kpn*I sites, respectively (Table S2), was subcloned into the pENTRTM1A plasmid (A10462; ThermoFisher, Waltham, MA, USA). The resulting donor plasmid was mixed with the pEarleyGate 104 destination vector to obtain the YPF-LjAFB6 fusion [72].

### 4.5. Quantitative Real-Time qRT-PCR

qRT-PCR was performed with a DNA Engine Opticon 2 System, MJ Research (Waltham, MA, USA) using SYBR to monitor dsDNA synthesis. The procedure is described in [73]. The *UBIQUITIN* (*UBI*) gene (AW719589) was used as an internal standard. The oligonucleotides used for the qRT-PCR are listed in Table S2.

### 4.6. LORE1 Lines Analyses

LORE1 lines 30003519 and 30004527 were obtained from the LORE1 collection [43,44,45]. Plants in the segregating populations were genotyped, and expression of homozygous plants tested with oligonucleotides listed in the Table S2. After PCR genotyping, shoot cuts of the homozygous plants were cultured and amplified in axenic conditions as described in [74].

### 4.7. Histochemical β-Glucuronidase (GUS) Analysis

Histochemical GUS was performed as described in [75]. Sections 80 μm thick were obtained with the vibratome (Leica VT1000S; Wetzlar, Germany) as reported in [76].

### 4.8. Confocal Imaging

Confocal microscope analyses were performed using a LeicaDMi8 (Leica Biosystems, Wetzlar, Germany) laser scanning confocal imaging system. For YFP detection, the excitation was at 488 nm and detection was between 515 and 530 nm. For chlorophyll detection, excitation was at 488 nm and detection over 570 nm.

### 4.9. Statistical Analyses

Statistical analyses were performed using the VASSARSTATS two way factorial ANOVA for independent samples program (http://vassarstats.net/ (accessed on 5 July 2021).

### 4.10. Phylogenetic Study

Evolutionary history was inferred using the Neighbor-Joining method [39], the optimal tree having a sum of branch length = 8.90683588. The percentage of replicate trees in which the associated taxa clustered together in the bootstrap test (500 replicates) are shown next to the branches [77]. The evolutionary distances were computed using the JTT matrix-based method [78] and given in the units of the number of amino acid substitutions per site. The analysis involved 50 amino acid sequences. All positions with less than 65% site coverage were eliminated. That is, fewer than 35% alignment gaps, missing data, and ambiguous bases were allowed at any position. There were a total of 564 positions in the final dataset. Evolutionary analyses were conducted in MEGA7 [79]. Sequence data for the proteins described in the phylogenetic tree shown in Figure 1 can be found at the following databases: *L. japonicus*, https://lotus.au.dk/ (accessed on 1 April 2021); *S. lycopersicon*, ftp://ftp.solgenomics.net/tomato_genome/annotation/ITAG4.0_release/ (accessed on 1 April 2021); *A. thaliana*, https://www.arabidopsis.org (accessed on 1 April 2021); *M. truncatula*, https://www.genome.jp/kegg-bin/get_htext?mtr00001 (accessed on 1 April 2021); *O. sativa*, http://rice.plantbiology.msu.edu/index.shtml (accessed on 1 April 2021); *V. vinifera*, http://www.plantgdb.org/VvGDB/ (accessed on 1 April 2021); *P. trichocarpa*, http://www.plantgdb.org/PtGDB/ (accessed on 1 April 2021).

### 4.11. Protein Modeling

The model was obtained online at the SWISS MODEL server, (swissmodel.expasy.org, accessed on 1 April 2021) by using tools of the Deep view Swiss PDB Viewer program [80]. The picture was generated by PyMOL program (Schrödinger, Inc., New York, NY, USA).

## Data Availability

Sequence data for the genes described in this article can be found in the Lotus database https://lotus.au.dk/ (accessed on 1 July 2021).

## Supplementary Materials

The following are available online at https://www.mdpi.com/article/10.3390/ijms22168495/s1. Figure S1. (A) The LjAFB6 amino acid sequence. Conserved amino acid residues are highlighted (red, F-box; green, conserved auxin binding residues; blue, residues involved in the oligomerization). (B) Three-dimensional structure model obtained using as template the X-ray structure of *A. thaliana* TIR1 (PDB code:2P1M) [37]. F-box and LRRs are indicated in red and blue, respectively. The model was obtained online at the SWISS MODEL server by using tools of the Deep view Swiss PDB Viewer program (Guex and Peitsch, 1997). The picture was generated by PyMOL program. Figure S2. Relative expression levels of the TIR1/AFB genes in different organs, normalized to the tissue with the highest transcript abundance. The heatmap was generated using the ExpAt tool available online at https://lotus.au.dk/expat/ (accessed on 5 May 2021) [38]. Figure S3. *Lotus japonicus* AFB6 localization at the nucleus. The LjAFB6-YFP translational fusion was transiently expressed in protoplast of tobacco mesophyll cells under the control of the 35S promoter. (A) YFP nuclear localization (B) DAPI nuclear localization (C) Merged image of chlorophyll autofluorescence and DAPI-stained nucleus. White bar = 20 μm. Figure S4. Phenotypic characterization of *Ljafb6* plants. Representative images of wild type and *Ljafb6* plants at 20 days after transfer on 2,4-D media. (A) Roots of wild type and *Ljafb6-1* plants grown on B5 Gamborg medium without 2,4-D. (B) Roots of wild type and *Ljafb6-1* grown in the presence of 0.01 μM 2,4-D. (C) Roots of wild type and *Ljafb6-1* plants grown in the presence of 0.1 μM 2,4-D. (D) Same plants as in C. (E) Roots of wild type and *Ljafb6-2* plants grown in the presence of 0.1 μM 2,4-D. (F) Same plants as in E. Figure S5. Phenotypic characterization of *Ljafb6* plants. (A) Representative images of the roots of wild type and *Ljafb6-1* plants at 20 days after transfer on 0.5 μM IAA media. (B) Same plants as in A. (B) Etiolated wild type and *Ljafb6* seedlings maintained for 2 weeks in the dark. Plant different genotypes are indicated. Figure S6. Number of lateral roots formed in wild type and *Ljafb6* plants. Plants were scored at 20 days after transfer on media supplemented with IAA. Bars represent means and SE of measures from three experiments (12 plants per experiment per condition). Figure S7. Mean of lateral root elongation of inoculated plants. Wild type and *Ljafb6* plants were grown in a B5 derived medium without nitrogen sources and inoculated with *M. loti*. Roots length were scored at 3 weeks after inoculation. The different concentrations of IAA are indicated. Bars represent means and SE of measures from three experiments (15 plants per experiment per condition). Asterisks indicate significant differences with wild type measures. * *p* < 0.001. Table S1. List of the *L. japonicus TIR1/AFB* members. Amino acid length and identity values with the *A. thaliana* members are indicated (the significant peaks of identity are highlighted in bold). Table S2. Oligonucleotides used in the present work. References [37,38] are cited in the supplementary materials.
