# Supplementary material for: The Lotus japonicus AFB6 Gene Is Involved in the Auxin Dependent Root Developmental Program
Source: Int J Mol Sci. 2021 Aug 6;22(16):8495. doi: 10.3390/ijms22168495 (PMC8395167; doi:10.3390/ijms22168495)
Supplement: Supplementary file 1 [file ijms-22-08495-s001.zip › ijms-1230245-supplementary.pdf]

A

MECRIRKKESPEPNILNQASP**FPDEVLERVLGMVKSRKDRSSVSLVCKEWYNAERWSRRS** VFIGNCYSVSPEILTRRFPNIRSVTLKGKPR**F**SD**F**NLVPANWGADI
RSWLVVFADKYPLLEELRL**K**RMT**V**SDESLEFLALSFPYFKALSLLS**C**D**GF**STD**G**LAA**I**ATSCKNLTELDIQENGID**D**KSGNWLSCFPESFTSLEVLNFANLPNDVN
SDALEKLVSRCKSLKTLKVNRSVTLEQLQRLLVRAPQLCELGSGSFSEDDLTAQQYSEIESAFNNCKNLHTLSGLWDPAEQYLPLLYPACTNLTFFNLSYAPLDGD
DLAKLLVHCPNLRRLWVVDTVEDKGLEAVGSHCPLLEELRVFPADPFEDEAIGHGVTESGFVALSRGCPRLHYVLYFCRQMTNAAVATVVQNCPDFTHF **R**LCIMNP
GQPDYLTHEPMDEAFGAVVKTCSKLQ**R**LAVSGLLTDLTFEYIGKYAKNLETL**S**V**A**FAGSSDWGMECVLRG**C**PKLRK**LE**IRDCPFGNAALLSGLERYESMRSLWMSD
CKLTMNGCRLLAKEMPRLNVEVIKEEGCYEGPAQRVYVYRSVAGPRRDAPSFVLAHCGPHNARGSGKGSTTWSIVCTMQPYLVCYMRGYFQNL

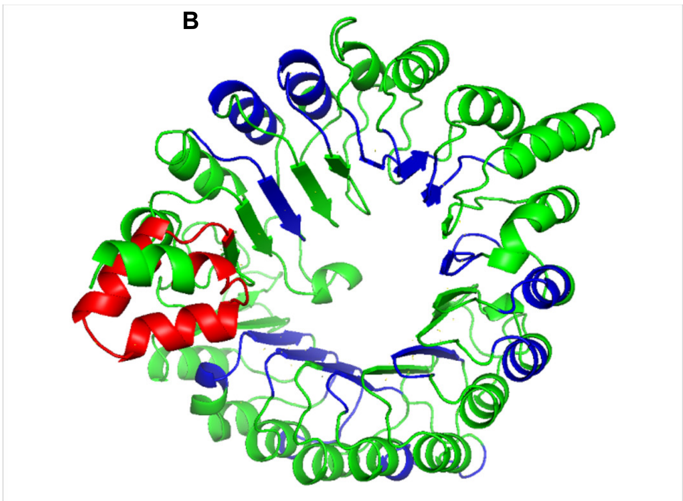

Figure S1. (**A**) The LjAFB6 amino acid sequence. Conserved amino acid residues are highlighted (red, F-box; green, conserved auxin binding residues; blue, residues involved in the oligomerization). (**B**) Three-dimensional structure model obtained using as template the X-ray structure of *A. thaliana* TIR1 (PDB code:2P1M) [37]. F-box and LRRs are indicated in red and blue, respectively. The model was obtained online at the SWISS MODEL server by using the tools under Deep view Swiss PDB Viewer program (Guex and Peitsch, 1997). The picture was generated by PyMOL program.

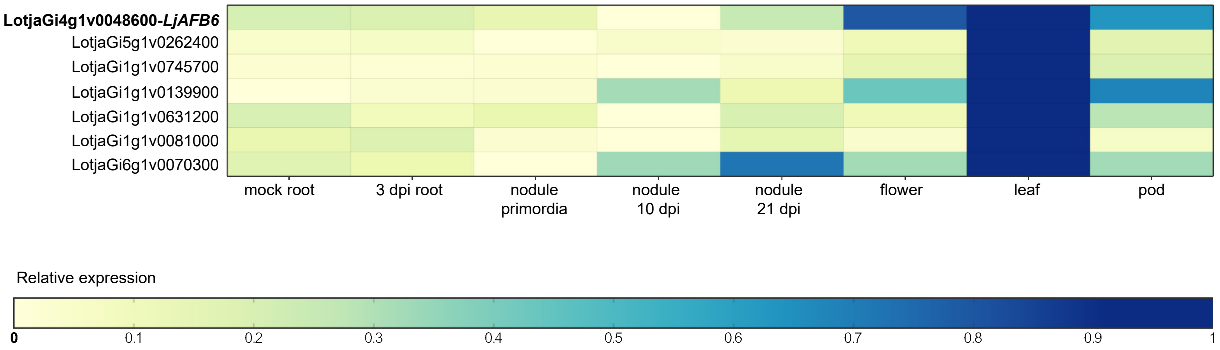

Figure S2. Relative expression levels of the TIR1/AFB genes in different organs, normalized to the tissue with the highest transcript abundance. The heatmap was generated using the ExpAt tool available online at https://lotus.au.dk/expat/ (accessed on 5 May 2021) [38].

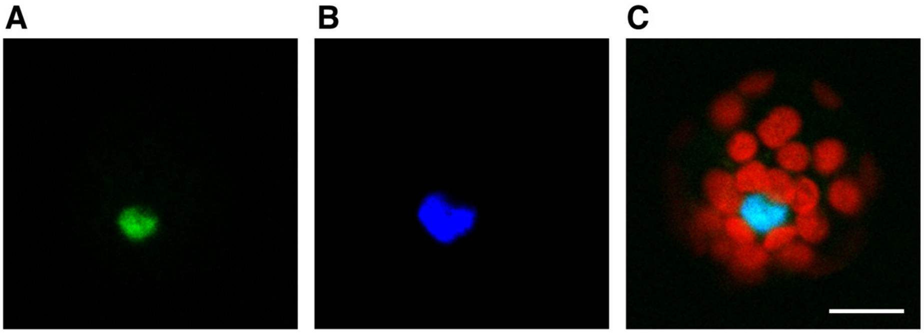

Figure S3. *Lotus japonicus* AFB6 localization at the nucleus. The LjAFB6-YFP translational fusion was transiently expressed in protoplast of tobacco mesophyll cells under the control of the 35S promoter. (**A**) YFP nuclear localization (**B**) DAPI nuclear localization (**C**) Merged image of chlorophyll autofluorescence and DAPI-stained nucleus. White bar = 20 μm.

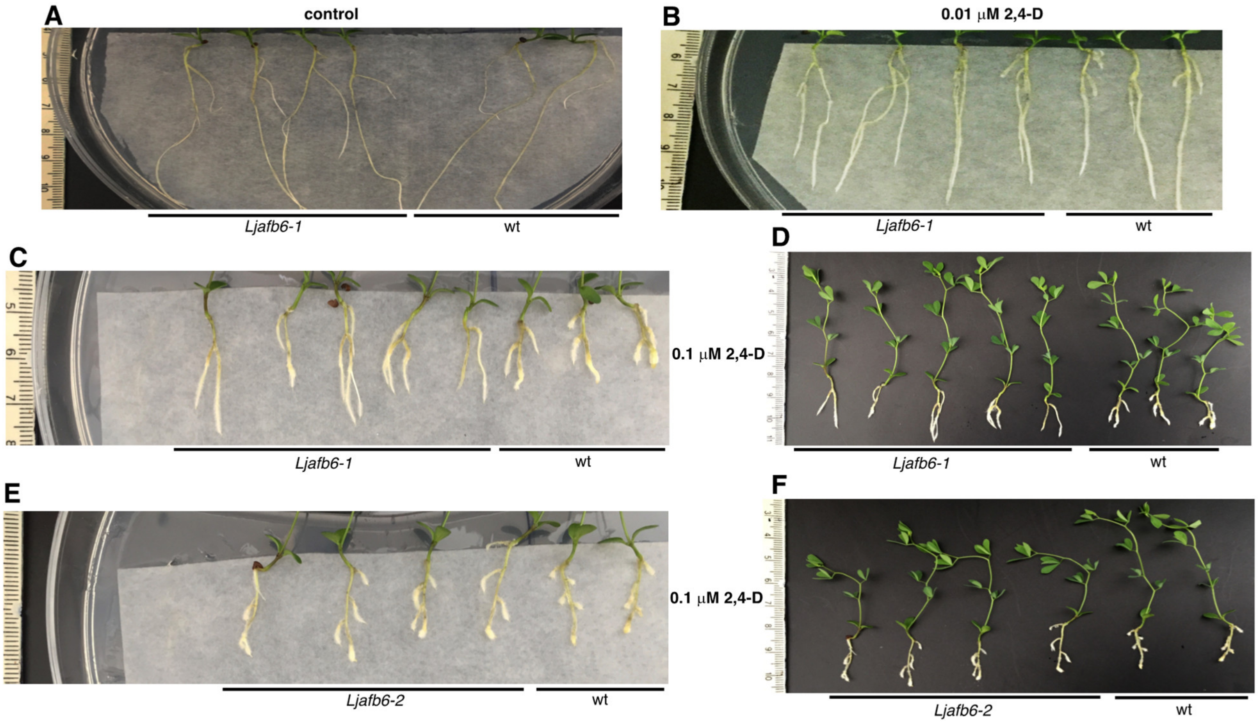

Figure S4. Phenotypic characterization of *Ljafb6* plants. Representative images of wild type and *Ljafb6* plants at 20 days after transfer on 2,4-D media. (**A**) Roots of wild type and *Ljafb6-1* plants grown on B5 Gamborg medium without 2,4-D. (**B**) Roots of wild type and *Ljafb6-1* grown in the presence of 0.01 μM 2,4-D. (**C**) Roots of wild type and *Ljafb6-1* plants grown in the presence of 0.1 μM 2,4-D. (**D**) Same plants as in C. (**E**) Roots of wild type and *Ljafb6-2* plants grown in the presence of 0.1 μM 2,4-D. (**F**) Same plants as in **E**.

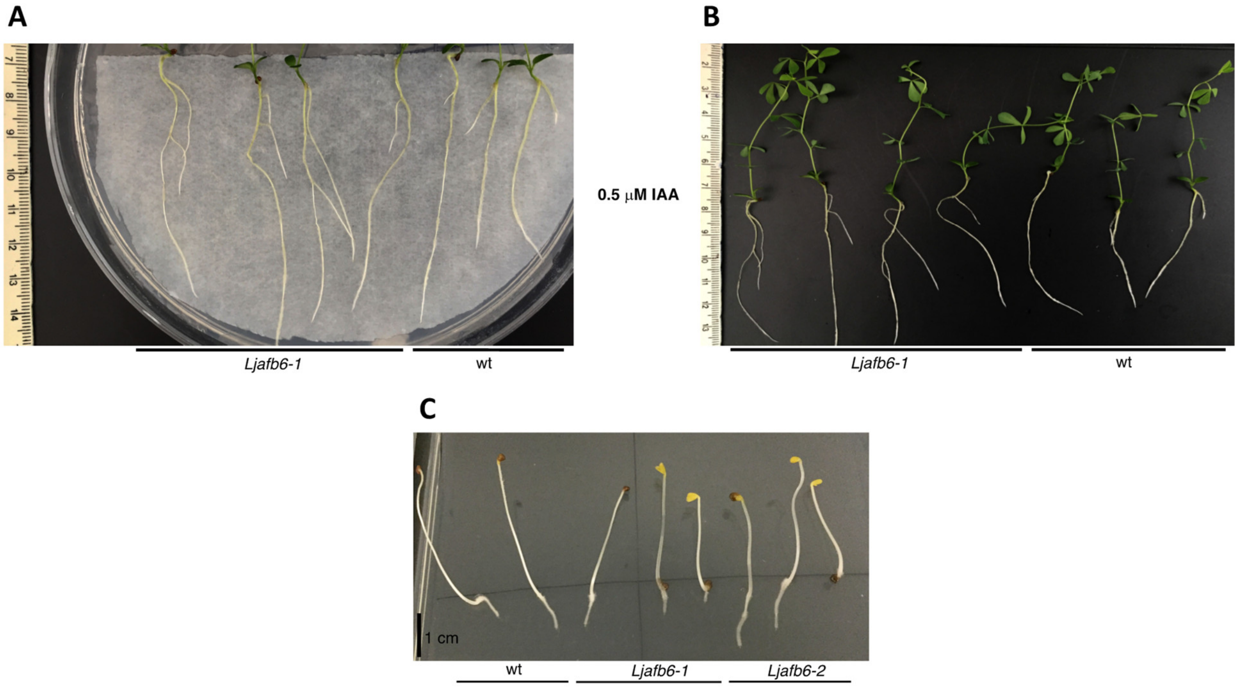

Figure S5. Phenotypic characterization of *Ljafb6* plants. (**A**) Representative images of the roots of wild type and *Ljafb6-1* plants at 20 days after transfer on 0.5 µM IAA media. (**B**) Same plants as in A. (**B**) Etiolated wild type and *Ljafb6* seedlings maintained for 2 weeks in the dark. Plant different genotypes are indicated.

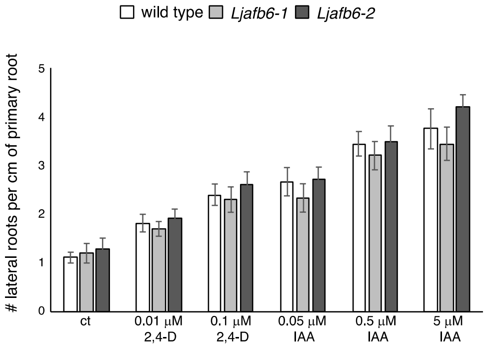

Figure S6. Number of lateral roots formed in wild type and *Ljafb6* plants. Plants were scored at 20 days after transfer on media supplemented with IAA. Bars represent means and SE of measures from three experiments (12 plants per experiment per condition).

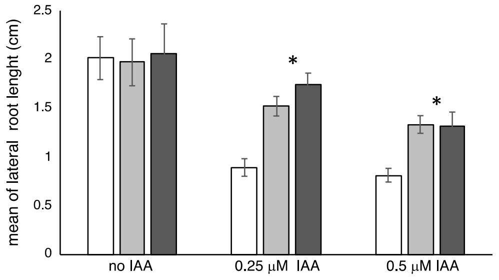

Figure S7. Mean of lateral root elongation of inoculated plants. Wild type and *Ljafb6* plants were grown in a B5 derived medium without N sources and inoculated with *M. loti*. Roots length were scored at 3 weeks after inoculation. The different concentrations of IAA are indicated. Bars represent means and SE of measures from three experiments (15 plants per experiment per condition). Asterisks indicate significant differences with wild type measures. * $p < 0.001$.

Table S1. List of the *L. japonicus TIR1/AFB* members. Amino acid length and identity values with the. *A. thaliana* members are indicated (the significant peaks of identity are highlighted in bold).

Gene	Aa Length	AtTIR1	AtAFB1	AtAFB2	AtAFB3	AtAFB4	AtAFB5	AtCOI1
LotjaGi4g1v0048600.1	623	56%	53%	54%	54%	49%	49%	33%
LotjaGi5g1v0262400.1	583	**78%**	67%	59%	61%	49%	51%	32%
LotjaGi1g1v0745700.1	585	**80%**	67%	61%	60%	49%	50%	32%
LotjaGi1g1v0631200.1	572	60%	56%	**74%**	**75%**	49%	51%	32%
LotjaGi1g1v0139900.1	631	50%	49%	50%	49%	67%	**72%**	29%
LotjaGi1g1v0081000.1	603	35%	35%	34%	34%	32%	33%	**72%**
LotjaGi6g1v0070300.1	590	34%	34%	34%	35%	32%	34%	**67%**

Table S2. Oligonucleotides used in the present work.

Promoter-gusA Fusion	
For-GUS-*Sal*I	CCGGTCGACATGAGGAGGAAGAAGGCAGG
Rev-GUS-*Bam*HI	CTAGGATCCAGATTCCTTCTTTCTGATCCT
LjAFB3-GFP fusion	
F-GFP-*Bgl*II	GAAGATCTATGGAGTGCAGGATCAGAAAG
R-GFP-*Kpn*I	GGGGTACCCAGGTTCTGGAAATAGCCTCT
qRT-PCR	
F-LotjaGi4g1v0048600(AFB3)	TTATCGTTCTGTTGCTGGCC
R-LotjaGi4g1v0048600.1(AFB3)	AGGTTCTGGAAATAGCCTCTCA
F-LotjaGi5g1v0262400	ACTTCAGGGTGTTGGTCCTG
R-LotjaGi5g1v0262400	TGGGGCACCTACTAACCAAG
F-LotjaGi1g1v0745700	GTGCAGGAGGAAAGTCTTCG
R-LotjaGi1g1v0745700	CTCAAGCCAAGGAAAAGCAC
F-LotjaGi1g1v0631200	ATAACGGTGGCCAAGAACTG
R-LotjaGi1g1v0631200	GAACACCTGGTCGGTCAACT
F-LotjaGi1g1v0139900	TCTGGTTTGCTGACTGATCG
R-LotjaGi1g1v0139900	TTGCAGTTCGACATCCAGAG
LORE1 lines genotyping	
Line 30003519	
F3519	CAACGCTGAGAGATGGTCAAGGAGGA
R3519	AGCACCCCCATCAAGCAGGAAACA
Line 30004527	
F4527	CAGCAGCATTGGTCATCTGACGGC
R4527	TGAACTTTGCCAACCTTCCCAATGA
ARF expression	
For-IAASynth1	ACTACACCAGCCCCAATGAG
Rev-IAASynth1	GGTCCAAGGTGCCTGTTTTA
For-IAASynth2	GCAATCCCCTCGTTCATCTA
Rev-IAASynth2	GTTGAATGTCAGGCAGCAGA
